# Investigation of the Effect of Magnification, Accelerating Voltage, and Working Distance on the 3D Digital Reconstruction Techniques

**DOI:** 10.1155/2020/3743267

**Published:** 2020-09-28

**Authors:** Seyed Mahmoud Bayazid, Nicolas Brodusch, Raynald Gauvin

**Affiliations:** Department of Materials Engineering, McGill University, Montreal, Quebec, Canada H3A 2B2

## Abstract

In this study, the effect of Scanning Electron Microscopy (SEM) parameters such as magnification (*M*), accelerating voltage (*V*), and working distance (WD) on the 3D digital reconstruction technique, as the first step of the quantitative characterization of fracture surfaces with SEM, was investigated. The 2D images were taken via a 4-Quadrant Backscattered Electron (4Q-BSE) detector. In this study, spherical particles of Ti-6Al-4V (15-45 *μ*m) deposited on the silicon substrate were used. It was observed that the working distance has a significant influence on the 3D digital rebuilding method via SEM images. The results showed that the best range of the working distance for our system is 9 to 10 mm. It was shown that by increasing the magnification to 1000x, the 3D digital reconstruction results improved. However, there was no significant improvement by increasing the magnification beyond 1000x. In addition, results demonstrated that the lower the accelerating voltage, the higher the precision of the 3D reconstruction technique, as long as there are clean backscattered signals. The optimal condition was achieved when magnification, accelerating voltage, and working distance were chosen as 1000x, 3 kV, and 9 mm, respectively.

## 1. Introduction

The development of the scanning electron microscope (SEM) [1] and the 3D digital reconstruction techniques [2–4] along with the development of image processing methods, hardware and software, can significantly help in a broad range of applications such as investigation of fracture surfaces [5], surface engineering [6], 3D printing [7], and biological researches [8]. To reconstruct a 3D digital image from multiple SEM images, correct types of features should be chosen on the 2D images [9]. Therefore, the quality of the SEM images is the critical point in any 3D digital reconstruction method. There is a large body of research in literature on 3D digital reconstruction techniques [10–12]; however, many problems remain unsolved related to the effect of SEM parameters on the quality of 3D digital images which are obtained with SEM micrographs. Recently, some efforts have been devoted to improving the quality and correctness of the 3D digital images which were obtained with secondary electron (SE) images [13–15]. The quality of the 3D digital reconstruction method is based on shape, surface structure of the object, and the SE micrograph quality [16]. It was shown that the in-lens detector has much better results of 3D image reconstruction in comparison with the Everhart-Thornley detector since the Everhart-Thornley detector is subject to shadowing effects more than the in-lens detector [17]. It is also reported that the SEM magnification does not improve 3D digital reconstruction results [4]. However, magnification can affect the resolution and the quality of the micrographs [18, 19], and as a result, it can affect the quality of 3D digital rebuilding. By taking into account that rotation and tilting are two main factors in 3D digital reconstruction of SEM images, it was observed that for the case of rotations, the largest uncertainty contribution is due to the reproducibility of the rotational angle, followed by the bias of the pixel size. On the other hand, for the case of tilting, the largest uncertainty contribution is due to the bias of the pixel size, followed by the reproducibility of the tilt angle [20]. On the other hand, there is very limited research concerning the 3D digital images which are obtained with backscattered electron (BSE) images [21, 22]. Since the presence of a rough surface differs significantly with the type, solid angle, and take-off angle of the detector used to collect the signal [23], the location of the detector could play a significant role for obtaining more signals from rough surfaces. Therefore, backscattered electrons are most efficiently and selectively collected with a 4-Quadrant Backscattered Electron (4Q-BSE) detector directly located on top of the sample. Consequently, in this paper, the effects of SEM parameters on the 3D digital images which are taken via BSE images, as the first step of the quantitative characterization of fracture surfaces, are studied.

## 2. Materials and Method

**Figure 1:**
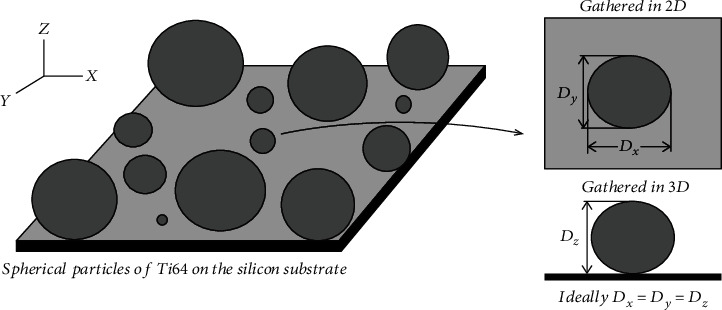
The spherical particles of Ti-6Al-4V on the silicon substrate.

**Figure 2:**
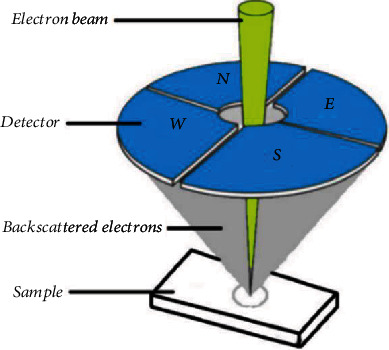
4Q-BSE detector.

The spherical particles of Ti-6Al-4V with different sizes (15-45 *μ*m) deposited on the silicon substrate were used (Figure 1). Given that the size of the spherical particles was available, the use of these spherical particles on a flat surface allowed ensuring the accuracy of the produced 3D digital images. The imaging process was performed with the SU3500 variable-pressure SEM with the 4Q-BSE detector (Figure 2) in the vacuum mode (30 Pa) with only one scan to find out the effect of SEM parameters such as magnification (*M*), accelerating voltage (*V*), and working distance (WD) in the range of 500-2000x, 3-20 kV, and 8-12 mm, respectively. Beam current and spot intensity were set to 146 *μ*A and 40 (unitless value from the SEM operating software), respectively. Each image was focused manually, and manual stigmator correction was applied after every image. The relative radius (*R*_r_) (Equation (1)) was used as a comparison operator for the size of any particles between 2D and 3D images. The relative radius was measured from 3D reconstruction's profile. After the 3D rebuilding of spherical particles, it was possible to take measurements from the roughness profile at any position in the 3D image and then make an average.

(1) $$\begin{array}{c} R_{\text{r}}=\frac{r_{Z}-r_{X=Y}}{r_{X=Y}}\times 100\; \end{array}$$

where *R*_r_ is the relative radius in percentage, *r*_*Z*_ is the radius of a spherical particle in the *Z* direction (in the 3D image), and *r*_*X*=*Y*_ is the radius of the same spherical particle in the *X* or *Y* direction (in the 2D image).

## 3. Results

**Figure 3:**
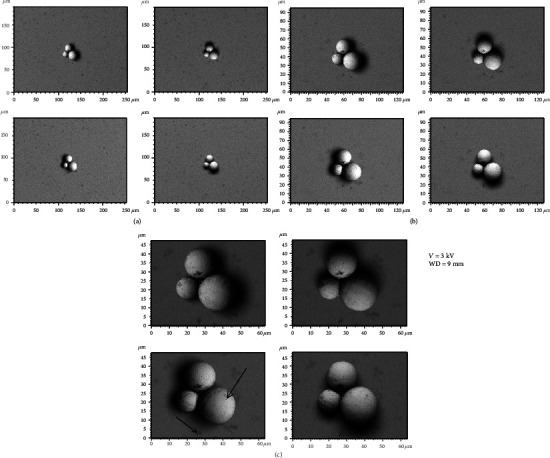
Backscattered electron images taken by the 4Q-BSE detector at different magnifications: (a) 500x, (b) 1000x, and (c) 2000x.

**Figure 4:**
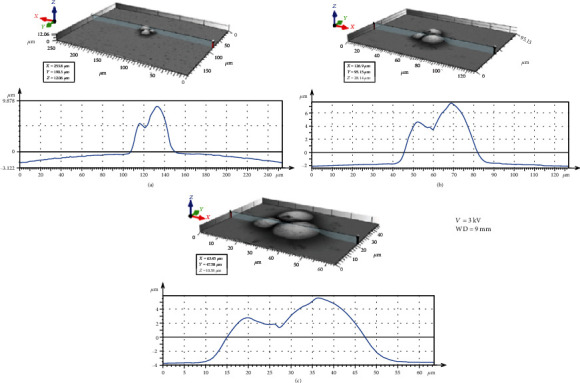
3D digital reconstructed image along with the profile at different magnifications: (a) 500x, (b) 1000x, and (c) 2000x.

**Figure 5:**
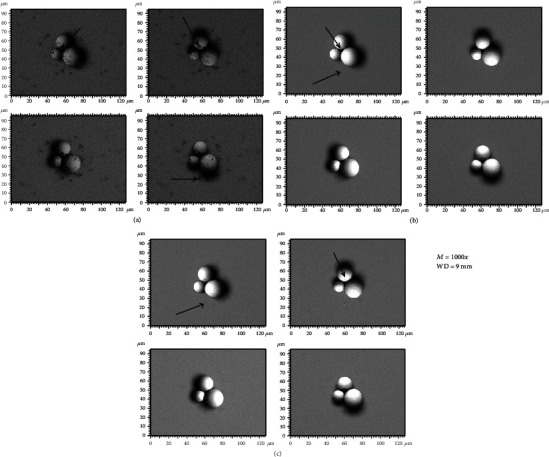
Backscattered electron images taken by the 4Q-BSE detector at different accelerating voltage: (a) 3 kV, (b) 10 kV, and (c) 20 kV.

**Figure 6:**
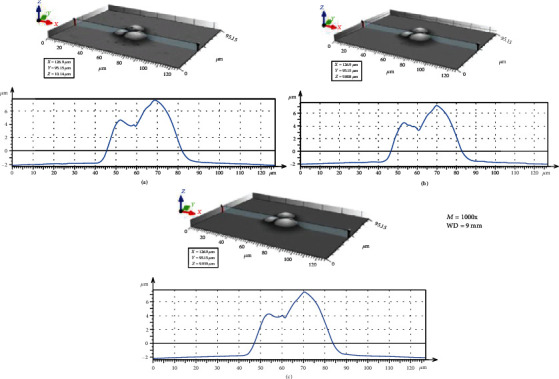
3D digital reconstructed image along with the profile at different accelerating voltage: (a) 3 kV, (b) 10 kV, and (c) 20 kV.

Figure 3 illustrates the BSE images of the different spherical particles with different magnifications. The accelerating voltage and working distance were set as constant values of 3 kV and 9 mm, respectively.  Figure 4 presents 3D digital reconstruction of Ti-6Al-4V particles along with the height profile which is shown in Figure 3.  Figure 4 shows that by increasing the magnification, the roughness profile which is the radius of the spherical particle in a 3D mode increases and becomes comparable with the radius of the spherical particle in a 2D mode. Therefore, the higher the magnification, the higher the precision of the 3D digital image.  Figure 5 shows the BSE images of the different spherical particles with different accelerating voltage. The magnification and working distance were set as constant values of 1000 kV and 9 mm, respectively.  Figure 5 shows that the appearance of shadow and small features (shown with arrows) in the 2D images diminishes by increasing the accelerating voltage because the interaction volume is proportional to the accelerating voltage (Equation (2)) [23]. The profile in Figure 6 shows the same trend; the higher the accelerating voltage, the lower the precision of the 3D digital image.

(2) $$\begin{array}{c} R_{K-O}\left(\text{nm}\right)=27.6\;{\left(\frac{A}{Z}\right)}^{0.89}\rho E_{0}^{1.67} \end{array}$$

where *A* is the atomic weight (g/mol), *Z* is the atomic number, *ρ* is the density (g/cm^3^), and *E*_0_ is the incident beam energy (keV).

**Figure 7:**
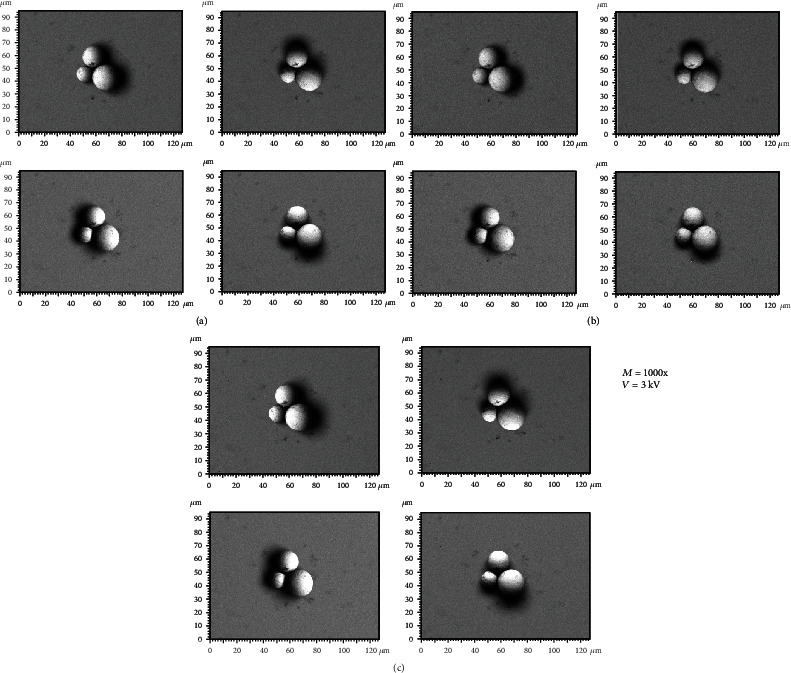
Backscattered electron images taken by the 4Q-BSE detector at different working distances: (a) 7 mm, (b) 9 mm, and (c) 11 mm.

**Figure 8:**
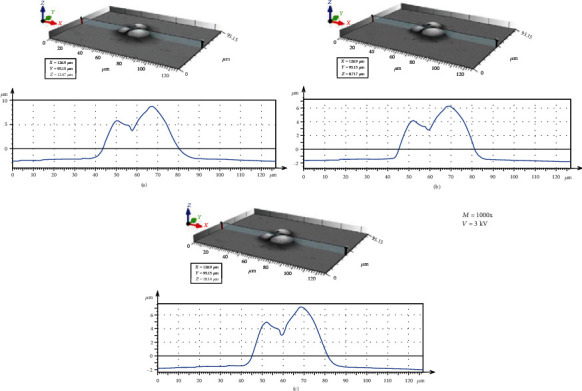
3D digital reconstructed image along with the profile at different working distances: (a) 7 mm, (b) 9 mm, and (c) 11 mm.

The BSE images of the different spherical particles which were taken at different working distances are shown in Figure 7. The magnification and accelerating voltage were set as constant values of 1000x and 3 kV, respectively. The profile in Figure 8 shows a different scenario; the precision of the 3D digital image increases by increasing the working distance, but after a specific point (WD = 9 mm), it starts to decrease.

**Figure 9:**
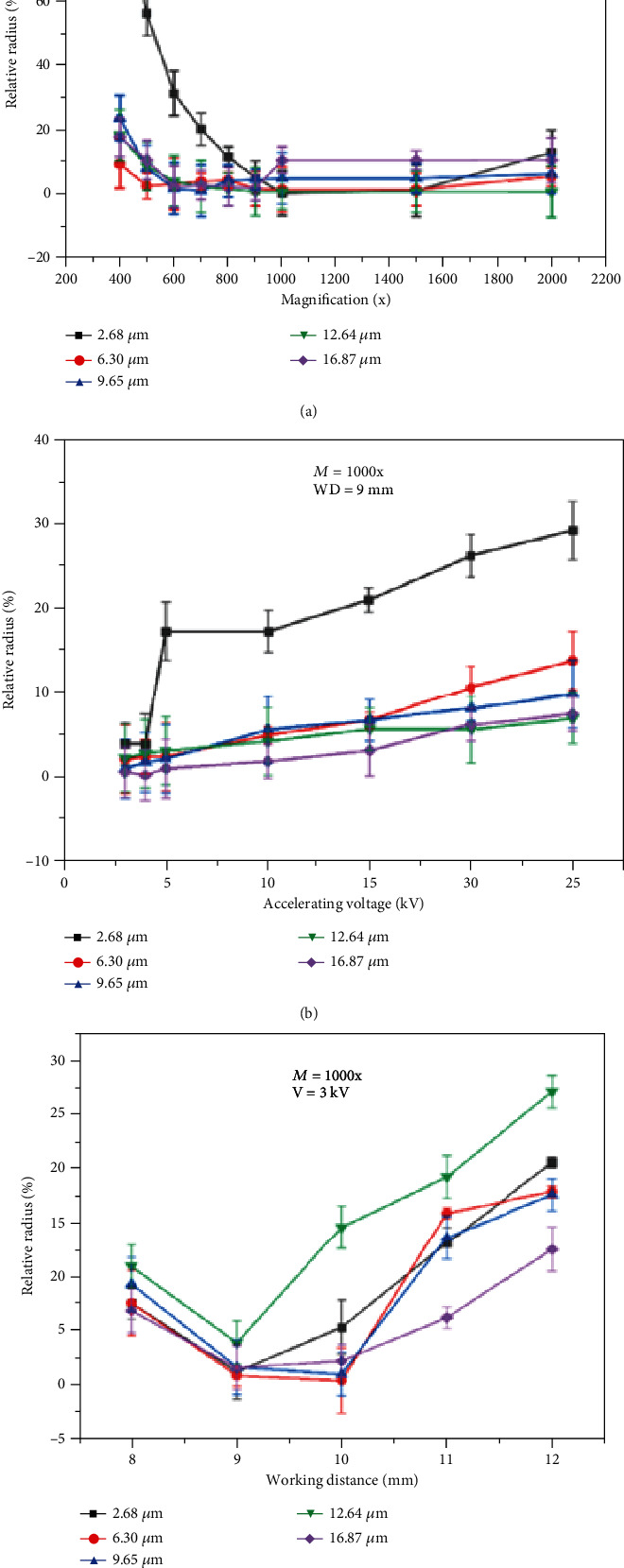
Relative radius versus (a) magnification, (b) accelerating voltage, and (c) working distance (each graph represents one particle).

Figure 9 shows the value of the relative radius as a function of magnification (*M*), accelerating voltage (*V*), and working distance (WD) for different sizes of particles, separately. Based on the relative radius (*R*_r_) (Equation (1)) (a comparison operator for the size of any particles between 2D and 3D images), to obtain high accuracy of 3D reconstruction data, we need to have small relative radius (*R*_r_) for any 3D rebuilding. It can be seen from Figure 9(a) that by increasing the magnification until 1000, the relative radius decreases. On the other hand, by increasing the magnification more than 1000x, a significant change in the relative radius is not seen. The result showed that when the magnification is less than 1000x, the small features such as small particles (2.68 *μ*m) cannot be seen even in the 2D images. Therefore, using these images for 3D reconstruction is not useful and makes a lot of error (Figure 9(a)). One way to solve this problem could be using the low accelerating voltage in order to decrease the interaction volume. Regardless of the size of the spherical particles, as the accelerating voltage rises, the relative radius enhances, which is not appropriate for 3D reconstruction, because the size of the interaction volume increases with the accelerating voltage (Equation (2)) [23]. In this situation, signals (BSE) will come from deeper inside of the sample, and consequently, the resolution of small features on the surface will decrease. Therefore, at low accelerating voltage, the interaction volume is small and close to the surface, and subsequently, obtaining more signals to produce a high-resolution image is possible. Moreover, the experimental results showed that reducing the accelerating voltage more than 3 kV would result in disruptive results due to losing signals without enough energy in reaching the surface (Figure 9(b)). Then, Figure 9(b) shows that when the accelerating voltage increases from 3 to 20 kV, *R*_r_ (which accounts for the amount of error) increases from the range of 0-5% to the range of 10-15%, which is not appropriate for 3D reconstruction. On the other hand, Figure 9(c) displays that the relative radius declines as the working distance increases, but larger working distance causes the relative radius to worsen.

## 4. Discussion

It was observed (Figures 4 and 9(a)) that by increasing the magnification to 1000, the relative radius declines thereby increasing the quality of 2D images. However, by increasing the magnification over 1000x, there is no significant improvement on the quality of 2D images and the relative radius. This behavior can be explained by the size of the pixels at any image. For any combination of SEM parameters, there is always a threshold contrast below which features of the specimen will not be visible. This threshold contrast depends on the relative size and shape of the feature of interest. The visibility of large objects and extended linear objects persists when small objects have dropped below the visibility threshold. That is, at low magnification, the size of the pixel increases. Each pixel represents a unique sampling of specimen features and properties, provided that the signals collected are isolated within the area represented by that pixel [18]. Therefore, at low magnification (*M* < 1000x), the specimen features and properties cannot be seen, and as a result, the relative radius is too large. In addition, this trend worsens for features and properties of the specimen which are very small (Ti-6Al-4V particle with a 2.68 *μ*m radius) at low magnification. From these curves (Figure 9(a)), the relative radius and hence the accuracy of 3D images produced are a strong function of the magnification of the taken picture and these effects must be included in the digital 3D reconstruction method.

Observation showed that (Figures 6 and 9(b)), regardless of the size of the spherical particles, as the accelerating voltage rises, the relative radius enhances, which is not appropriate for 3D reconstruction. The size of the interaction volume increases rapidly as the accelerating voltage increases [18]. In this situation, (BSE) signals will come from deeper inside of the sample, and consequently, the resolution of small features on the surface will decrease. Therefore, at low accelerating voltage, the interaction volume is small and close to the surface, and subsequently, obtaining more signals to produce a high-resolution image is possible. Moreover, the experimental results showed that by dropping the accelerating voltage more than 3 kV, it would result in a disruptive outcome due to losing signals without enough energy in reaching the surface. Note that this is true for a thermionic gun due to the spherical aberration, but this might not be true with a FE-SEM. Figures 8 and 9(c) display the behavior of the 3D digital reconstruction method and relative radius when the working distance changes. There are two scenarios: first, for high depth of field, the largest working distance is needed, and second, for a high-resolution mode, the smallest working distance is needed [18]. When considering high resolution, as well as high depth of field, necessary to obtain a high-quality image with more features, the behavior of the curves (see Figure 9(c)) is sensible. More specifically, reaching the high depth of field requires the largest working distance. On the other hand, high resolution requires the smallest working distance. The largest working distance is mandatory if we want to have a high depth of field. On the other hand, the smallest working distance is necessary if we need high resolution. As a result, the pattern of the curves (see Figure 9(c)) makes sense. Note that the increase in the working distance more than the optimum range (9-10 mm) causes a decrease in the resolution.

## 5. Conclusion

The present study was aimed at identifying the optimal and most influential SEM parameters on the accuracy of the 3D digital reconstruction method which is under development. Various combinations of processing parameters were considered to evaluate the relative importance of parameters. It was observed that the working distance is a significant influential parameter in the digital 3D rebuilding method via SEM images. The best condition was obtained when the working distance was between 9 and 10 mm. It was found that by increasing the magnification to 1000x, the quality of 2D images which is important for 3D digital reconstruction improved. However, for magnifications over 1000x, not only is there no significant improvement in the quality of 2D images but also it can reduce the scan size. From our observations, as the accelerating voltage increases (more than 3 kV), the appearance of the small feature in the 2D images decreases due to the size of the interaction volume, which is not appropriate for 3D reconstruction. SEM parameters including magnification at 1000x, accelerating voltage at 3 kV, and working distance at 9 mm have been found to be the optimal parameters for the specific geometry of our SEM with a BSE detector located on top of the sample surface. It must be noted though that due to varying detector-pole piece distances available in commercially available SEMs, these optimum values may vary slightly and may be optimized as well for each instrument.

## Data Availability

The data that support the findings of this study are available from the corresponding author (Seyed Mahmoud Bayazid, email: mahmoud.bayazid@mail.mcgill.ca) upon reasonable request.
